# Echocardiographic evaluation of left ventricular systolic function by the M‐mode lateral mitral annular plane systolic excursion in patients with Duchenne muscular dystrophy age 0‐21 years

**DOI:** 10.1002/hsr2.188

**Published:** 2020-10-05

**Authors:** Melissa K. Webb, Poonam P. Thankavel, Claudio Ramaciotti

**Affiliations:** ^1^ Department of Pediatrics University of Texas Southwestern Medical Center Dallas Texas

**Keywords:** cardiomyopathy, transthoracic echocardiography

## Abstract

**Background and Aims:**

Duchenne muscular dystrophy (DMD) results in cardiac fibrosis and dysfunction. These patients frequently have poor image quality. Mitral annular plane systolic excursion (MAPSE) is a reproducible and reliable method for determining function and can be a valuable tool in patients with poor images. Our study was performed to evaluate the feasibility of MAPSE and compare it to shortening fraction (SF) in patients with DMD.

**Methods:**

Lateral M‐mode MAPSE was obtained on all echocardiograms performed on DMD patients aged 0 to 21 years between October 2013 and April 2015. Retrospectively, interobserver and intraobserver variability was determined for these measurements and each measurement was compared to patient characteristics and measured values of SF.

**Results:**

There was good interobserver (*r* ^2^ = .66, *P* = .0081) correlation. Seventeen of 59 echocardiograms (29%) had abnormal SF while 32 (54%) echocardiograms had an abnormal M‐mode lateral MAPSE *Z*‐score. There was no significant association between lateral MAPSE *Z*‐score and SF. Age at the time of echocardiogram and time from diagnosis to echocardiogram both had a significant negative correlation with lateral MAPSE.

**Conclusions:**

Lateral M‐mode MAPSE measurements are reproducible in young patients with Duchenne muscular dystrophy. M‐mode lateral MAPSE may worsen over length of time with Duchenne muscular dystrophy. Further studies are necessary to provide absolute conclusions, but this study shows that lateral M‐mode MAPSE may be a valuable additional tool at routine echocardiogram in these patients.

## 1 INTRODUCTION

Duchenne muscular dystrophy (DMD) is an X‐linked recessive disorder that consists of a deficiency in the dystrophin gene. Dystrophin links actin to the sarcolemma, making it critical for muscle membrane stability and the contraction‐relaxation process. Progressive membrane damage, muscle necrosis, and muscle fibrosis eventually occur, resulting in cardiac dysfunction and poor long‐term cardiovascular outcomes.^1^, ^2^, ^3^ Shortening fraction (SF) and ejection fraction (EF) measured by echocardiography are routinely used to monitor cardiac function. However, many patients with DMD have poor acoustic windows making accurate evaluation of left ventricular systolic function difficult, since SF and EF measurements require adequate visualization of the endocardial border.^4^ While subjective evaluation of left ventricular function by experienced echocardiography readers has been validated,^5^ an objective parameter is helpful for guiding medical treatment.

Mitral annular plane systolic excursion (MAPSE) evaluates left ventricular longitudinal shortening and has been shown to correlate well with EF in adult and pediatric patients.^6^, ^7^ It is a quantitative measurement with reproducible results in both children and adults^6^, ^7^ and does not require high imaging quality for accurate measurements due to the high echogenicity of the mitral annulus.^8^ In fact, it has been recommended as a measurement in adult patients with poor acoustic windows in whom SF and EF cannot be obtained.^9^ Our study sought to determine the feasibility and value of MAPSE in the evaluation of patients age 0 to 21 years with DMD.

## 2 METHODS

This study is a retrospective analysis of clinically indicated echocardiograms and M‐mode lateral MAPSE measurements between October of 2013 and April of 2015, assessing the MAPSE in patients with Duchenne muscular dystrophy. The study also sought to evaluate any correlation between patient characteristics and SF with lateral M‐mode MAPSE measurements. This study was approved by the University of Texas Southwestern Internal Review board. Informed consent was obtained from patients and/or their guardians.

### 2.1 Subjects

Patient‐enrollment criteria included the following: age 0 to 21 years and diagnosis of Duchenne muscular dystrophy confirmed by skeletal muscular biopsy or genetic analysis. The echocardiograms were obtained as part of routine clinical assessment and not for the purpose of this study. Exclusion criteria included the presence of other hemodynamically significant cardiac abnormalities not typically encountered in patients with Duchenne muscular dystrophy (ie, ectopy, arrhythmias, heart block, and thinning of the left ventricle wall).^10^

### 2.2 Demographics

Patient data, including age, height, weight, body surface area, medications, age at diagnosis, and comorbid conditions were recorded.

### 2.3 Echocardiograms and analysis

Routine transthoracic echocardiograms were performed and lateral MAPSE was measured by a blinded reader in the four‐chamber apical view via M‐mode across the mitral valve annulus corresponding to the lateral left ventricular wall segments.

**Figure 1:**
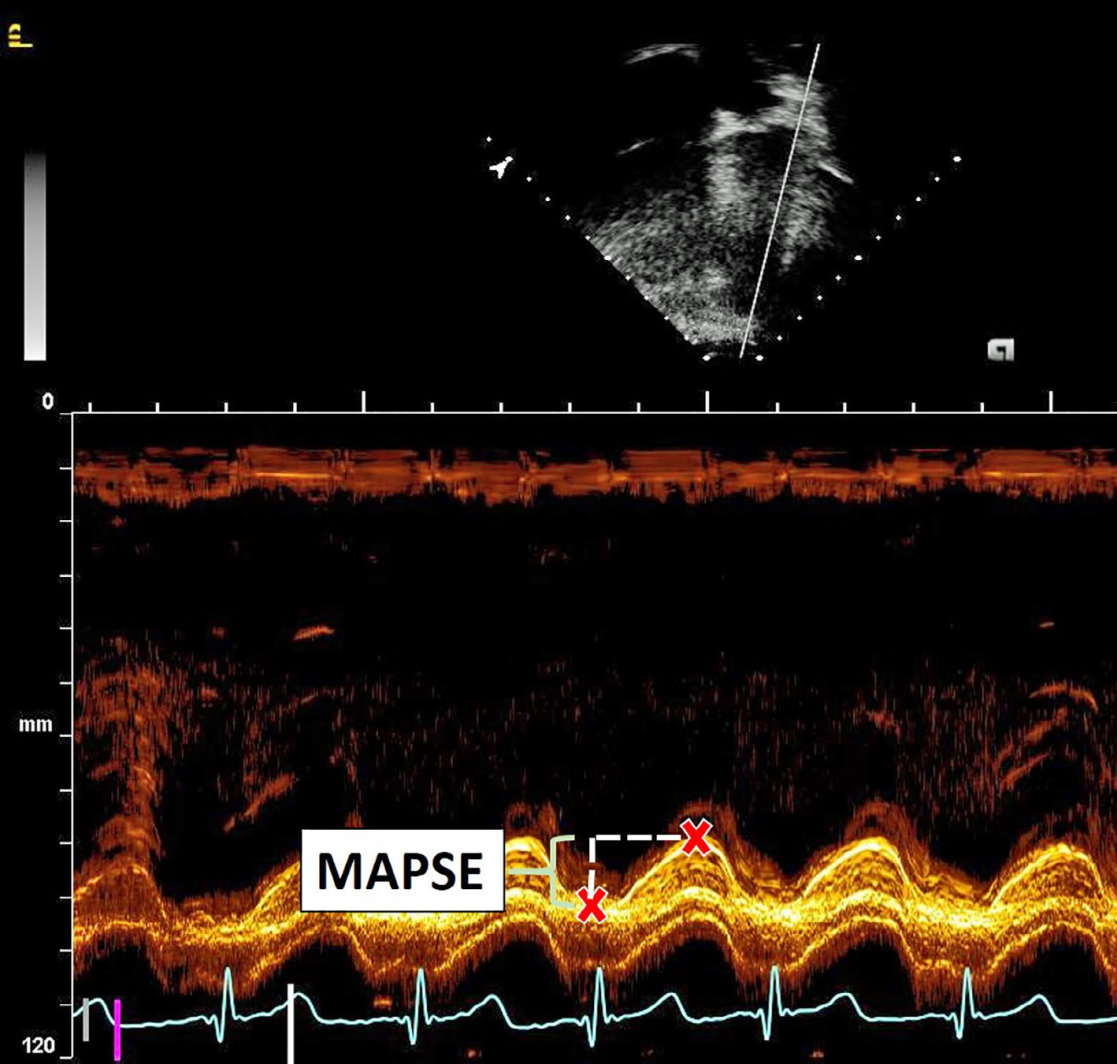
Four chamber echocardiographic image M‐mode across the lateral edge of the mitral valve annulus. The red letter “x” marks the position of the lateral wall at end diastole and at its highest point at the time of mitral valve closure. The height between these two positions is MAPSE. MAPSE, mitral annular plane systolic excursion; mm, millimeter

The distance from the lowest point at end‐diastole to the highest point during mitral valve closure was measured to demonstrate the systolic excursion of the mitral annulus (Figure 1). A second blinded reader performed each measurement for 10 patients to calculate interobserver variability. A three‐beat average was obtained and recorded for each measurement. Left ventricular SF was measured by M‐mode in the short axis at the level of the papillary muscles. Using previously described cut offs, SF was determined to be abnormal and assigned a 1 if the value was equal to or less than 28% and determined to be normal with an assigned number of 0 if the value was greater than 28%.^11^, ^12^

For those subjects between the ages of 0 and 19 years, lateral MAPSE *Z* values were determined based on previously described *Z* values.^13^ A *Z* score value < −2 was considered abnormal.

### 2.4 Statistical analysis

Interobserver and intraobserver variability was calculated using the coefficient of determination of Pearson correlation coefficients and paired *t* test respectively. Pearson correlation coefficients and coefficient of determinations were computed to examine the correlation between SF vs each actual MAPSE value, age at diagnosis, and time from diagnosis to echocardiogram. Chi‐square analysis was performed to determine any association between lateral MAPSE and binary SF values.

## 3 RESULTS

Fifty‐six patients and 67 echocardiograms were included in the study. The median age at echocardiogram was 12 years (1‐20 years). The median age at diagnosis was 5 years (0‐16 years). The median time from diagnosis to echocardiogram was 8 years (0‐16 years). All but one patient had genetic confirmation of DMD. Thirty six patients (64%) were on heart failure medications, consisting of an angiotensin converting enzyme inhibitor, beta blocker, and/or spironolactone. Forty patients (71%) were on prednisone. Not all patients on heart failure medications were also on prednisone.

Ten MAPSE measurements were obtained by two separate blinded observers and compared using a coefficient of determination (*r* ^2^) of the Pearson correlation coefficient (*r*) to determine interobserver variability. Significant correlation in the lateral MAPSE measurement was seen (*r* ^2^ = .66, *P* = .0081) between the two observers. There was no significant difference between intraobserver measurements when 10 measurements from the same patient and same observer were compared (mean difference −0.004 ± 0.073, *P* = .860). Coefficient of determination (*r* ^2^) of the Pearson correlation coefficient for absolute M‐mode lateral MAPSE value and SF was 0.18 (*P* = .0003).

Forty‐five patients and 59 echocardiograms were performed in patients 18 years old or less. These M‐mode lateral MAPSE measurements were therefore able to be standardized by previously defined *Z*‐score values.^13^ The M‐mode lateral MAPSE *Z* score tended to be abnormal more frequently than the shortening fraction. Seventeen of 59 echocardiograms (29%) had abnormal SF while 32 (54%) echocardiograms had an abnormal M‐mode lateral MAPSE (Table 1). There was no significant association between lateral MAPSE *Z*‐score and SF by Chi‐Square analysis. Of the 32 echocardiograms with an abnormal M‐mode lateral MAPSE *Z* score, 21 (66%) had a normal SF. There was no significant difference in age between those with an abnormal *Z* score with normal SF and those with an abnormal SF (*P* = .9).

**Table 1:** Number of echocardiograms by lateral MAPSE *Z* score measurement and shortening fraction

	*Z* score <−2	*Z* score >−2	Total
Normal shortening fraction	21 (35%)	21 (36%)	42 (71%)
Abnormal shortening fraction	11 (19%)	6 (10%)	17 (29%)
Total	32 (54%)	27 (46%)	59 (100%)

*Note*: There was a trend toward MAPSE measurements being more frequently abnormal than shortening fraction measurements.

Abbreviation: MAPSE, mitral annular plane systolic excursion.

Both time from diagnosis to echocardiogram and age at the time of echocardiogram had significant negative correlations to both MAPSE and SF (Table 2).

**Table 2:** Coefficient of determination of negative Pearson correlation coefficients for time since diagnosis

	*r* ^2^	*P*
Time from diagnosis to echo (MAPSE)	.140	.0019
Age at time of echo (MAPSE)	.063	.04112
Time from diagnosis to echo (SF)	.257	.0001
Age at time of echo (SF)	.139	.0017

*Note*: Time from diagnosis and age at the time of echocardiogram had significant negative correlations with both M‐mode lateral MAPSE and shortening fraction.

Abbreviations: MAPSE, mitral annular plane systolic excursion; SF, shortening fraction.

*P ≤ .05 is significant.

## 4 DISCUSSION

Our study demonstrated that MAPSE measurements can be obtained in a reproducible manner in a population with DMD and also that the M‐mode lateral MAPSE tended to be abnormal more frequently than SF. However, our data showed a low correlation between lateral M‐mode MAPSE and SF. It is not known the factors that determine different rates of progression of cardiac dysfunction among patients with Duchenne muscular dystrophy. More striking, brothers with identical mutations can have dissimilar rates of progression and responses to therapy.^14^ MAPSE measurements evaluate shortening of left ventricular longitudinal fibers and the poor correlation could be related to different responses to medications or different rates of progression of dysfunction of the longitudinal fibers. The finding of abnormal function by MAPSE with normal SF emphasizes that possibility. Our study was not designed to answer this question but it raises the possibility that MAPSE can be an adjunct in evaluation of left ventricular function in DMD.

Overall, this may indicate that the lateral MAPSE *Z* score provides an earlier indicator of abnormal function compared to SF, and therefore might demonstrate an abnormal value in patients who may not otherwise demonstrate an abnormal SF. Additional support for this possibility is that both time from diagnosis to echocardiogram and age at the time of echocardiogram negatively correlated with lateral M‐mode MAPSE. In other words, this correlation may indicate that the longer a patient has the diagnosis with time to develop cardiac fibrosis, the higher the possibility that the lateral M‐mode MAPSE will be diminished. In order for SF to become abnormal, it requires a longer time from diagnosis to echocardiogram and presumably, worse cardiac fibrosis. This suggestion is in agreement with the study by Duboc et al that supports placing patients on angiotensin converting enzyme inhibitors before documentation of an abnormal left ventricular function by echocardiography.^15^ In opposition to this theory is that there was no difference in age between those patients with a normal SF/abnormal MAPSE and abnormal SF/abnormal MAPSE. This may be due to low numbers in our study and certainly indicates that our data is not conclusive of abnormal lateral MAPSE as early evidence of poor function.

We found high inter and intraobserver correlation, indicating that the measurements are accurate and reproducible by multiple investigators. This is consistent with previous reports of ease of technique.^7^, ^9^ These findings suggest a role for lateral M‐mode MAPSE in assessing patients with Duchenne muscular dystrophy. The best treatment strategy in this population has not been defined. The finding of an abnormal lateral M‐mode MAPSE in the presence of normal ventricular function assessed by SF might indicate the need for more aggressive medical treatment. Therefore, as MAPSE can be obtained in a reproducible manner even in patients with poor acoustic windows, it can be of assistance in decision making about drug therapy for cardiac dysfunction.

A recent prospective multicenter controlled cross‐sectional study demonstrated that strain is frequently present despite normal left ventricle function in patients with Duchenne muscular dystrophy.^16^ Additionally, late gadolinium enhancement, indicating fibrosis by cardiac MRI, has been shown to occur early and be associated with progressive cardiac disease.^17^, ^18^ In fact, Tandon et al has demonstrated a 2.2 ± 0.31% per year decline in left ventricle EF when late gadolinium enhancement was present vs when it was not present.^18^ Due to its ability to detect early cardiac fibrosis, cardiac MRI with late gadolinium enhancement may be considered for routine testing in Duchenne muscular dystrophy patients.^17^, ^18^, ^19^, ^20^, ^21^ With further studies to verify its efficacy in this patient population, lateral MAPSE may similarly be considered for routine testing in Duchenne muscular dystrophy patients. A study evaluating lateral M‐mode MAPSE's relationship to strain or fibrosis found by late gadolinium enhancement on MRI may be of value.

## 5 LIMITATIONS

Limitations to this study include a small patient cohort in a single center study. The size of the study and single center nature limits the conclusions that can be made and the majority of findings are trends. In addition, lateral M‐mode MAPSE measures longitudinal function and may not account for dysfunction in other axes.

## 6 CONCLUSIONS

Our study demonstrates that in a population of patients with Duchenne muscular dystrophy, lateral MAPSE *Z* score was reproducible and tended to be abnormal more frequently than SF. This may indicate that lateral MAPSE is another indicator of cardiac dysfunction and might have a role in helping define the best treatment strategy for this population. Further studies are needed to confirm its value in detecting early cardiac dysfunction, its relationship to fibrosis, and for helping guide medical management.

## CONFLICT OF INTEREST

The authors have no conflict of interest to disclose.

## AUTHOR CONTRIBUTIONS

Conceptualization: Melissa K. Webb, Poonam P. Thankavel, Claudio Ramaciotti

Data Curation: Melissa K. Webb, Poonam P. Thankavel, Claudio Ramaciotti

Investigation: Melissa K. Webb, Poonam P. Thankavel, Claudio Ramaciotti

Methodology: Melissa K. Webb, Poonam P. Thankavel, Claudio Ramaciotti

Project Administration: Melissa K. Webb, Poonam P. Thankavel, Claudio Ramaciotti

Writing ‐ Original Draft Preparation: Melissa K. Webb, Poonam P. Thankavel, Claudio Ramaciotti

Writing ‐ Review & Editing: Melissa K. Webb, Poonam P. Thankavel, Claudio Ramaciotti

All authors have read and approved the final version of the manuscript.

Dr. Melissa Webb had full access to all of the data in this study and takes complete responsibility for the integrity of the data and the accuracy of the data analysis.

## TRANSPARENCY STATEMENT

Dr. Melissa Webb affirms that this manuscript is an honest, accurate, and transparent account of the study being reported; that no important aspects of the study have been omitted, and that any discrepancies from the study as planned have been explained.

## Data Availability

The data that support the findings of this study are available from the corresponding author upon reasonable request.
